# In Vitro Evaluation of Protected Black Soldier Fly Larvae (*Hermetia illucens*), Palm Oil, and Rice Bran Oil on Rumen Fermentation and Degradability

**DOI:** 10.3390/ani16213503

**Published:** 2026-11-06

**Authors:** Molthida Rungchaicharoenphai, Patcharida Sriwicha, Pheeraphong Phaengphairee, Anusorn Cherdthong, Sawitree Wongtangtintharn, Benjamad Khonkhaeng, Saroat Rawdkuen, Chanon Suntara

**Affiliations:** 1 Tropical Feed Resource Research and Development Center (TROFREC), Department of Animal Science, Faculty of Agriculture, Khon Kaen University, Khon Kaen 40002, Thailand; molthida.r@kkumail.com (M.R.); patcharida.s@kkumail.com (P.S.); perpan@kku.ac.th (P.P.); anusornc@kku.ac.th (A.C.); sawiwo@kku.ac.th (S.W.); 2 Department of Animal Science, Faculty of Agricultural Innovation and Technology, Rajamangala University of Technology Isan, Nakhon Ratchasima 30000, Thailand; benjamad.ko@rmuti.ac.th; 3 Food Science and Technology Program, School of Agro-Industry, Mae Fah Luang University, Chiang Rai 57100, Thailand; saroat@mfu.ac.th

**Keywords:** rumen fermentation, volatile fatty acids, degradability, Ca-soap, K-soap

## Abstract

This study evaluated the effects of protected and non-protected black soldier fly larvae oil (BSFLO), palm oil (PO), and rice bran oil (RBO) on ruminal fermentation characteristics and nutrient digestibility using an in vitro gas production technique. A completely randomized design with a 3 × 3 + 1 factorial arrangement was employed, consisting of three oil sources, three oil forms (non-protected, calcium soap, and potassium soap) supplemented at 4% of dry matter, and a control without oil. The results showed that oil supplementation without the basal diet reduced total volatile fatty acid (TVFA) production and ammonia nitrogen (NH_3_-N) concentration at 24 h, indicating an inhibitory effect of free fatty acids on rumen microorganisms. With the basal diet, TVFA concentrations at 24 h were higher in oil-supplemented treatments compared to the control. Specifically, protected BSFLO in both Ca-soap and K-soap forms showed the numerically highest TVFA concentrations (127.02 and 125.87 mmol, respectively), although these did not differ significantly from those of RBO Ca-soap and K-soap; BSFLO K-soap had a lower TVFA concentration than the control at 48 h. Oil supplementation significantly modified VFA profiles by decreasing the proportion of acetic acid, increasing propionic acid, and consequently lowering the acetate-to-propionate ratio. Ruminal pH was not affected by oil supplementation. While in vitro dry matter degradability was lower in the oil-supplemented treatments than in the control, IVDMD did not show differences between non-protected oil and protected oil treatments. Abomasal simulated digestion was not adversely affected by oil source or protection form. In conclusion, in the presence of a basal diet, BSFLO in Ca-soap and K-soap forms increased TVFA at 24 h, and BSFLO in all forms increased propionate proportions, without compromising rumen stability or abomasal simulated digestion under the conditions tested. These in vitro results indicate protected BSFLO’s potential as a lipid source in ruminant diets and warrant in vivo confirmation.

## Simple Summary

This study evaluated the effects of different oil sources and protection methods on rumen fermentation and degradability using in vitro techniques. BSFLO, PO, and RBO were tested in non-protected, Ca-soap, and K-soap forms. Oil supplementation modified VFA profiles by decreasing the proportion of acetic acid, increasing propionic acid, and consequently lowering the acetate-to-propionate ratio, and decreased IVDMD compared with the control group. In the presence of a basal diet, BSFLO in Ca-soap and K-soap forms increased TVFA at 24 h, and BSFLO in all forms increased the propionate proportion. Overall, insect-derived oil, especially BSFLO, shows potential as a sustainable feed additive for improving rumen fermentation in ruminants under in vitro conditions.

## 1. Introduction

Energy is one of the most costly nutritional components and plays a critical role in determining the productive efficiency of dairy cows, particularly in high-yielding animals or during early lactation [1], when cows repeatedly experience negative energy balance due to insufficient dietary energy intake [2]. Consequently, dairy rations widely incorporate dietary fats and oils because of their high energy density, allowing increased dietary energy without increasing feed intake [3]. However, lipid digestion and utilization in ruminants are considerably more complex than in monogastric animals. Dietary lipids are initially hydrolyzed in the rumen by lipolytic microorganisms, resulting in the breakdown of triglycerides into free fatty acids and glycerol [4]. These products may subsequently enter ruminal fermentation pathways or be utilized as energy sources by the animal [5]. Such transformations directly influence rumen fermentation patterns, the concentration and proportion of volatile fatty acids (VFAs), and overall nutrient digestibility [6].

Previous studies have demonstrated that moderate levels of oil supplementation can enhance dietary energy density without impairing rumen fermentation, provided that inclusion levels remain within an optimal range [7]. In general, supplementation at approximately 3–4% of dietary dry matter does not adversely affect microbial activity or fiber digestibility in the rumen [8]. In Surti goats fed rice bran oil (RBO) in their diet, an increase in propionate concentration and a decrease in acetate concentration were observed, resulting in a lower acetate-to-propionate ratio and reduced methane production [9]. Nevertheless, the effects of dietary oils on rumen fermentation are highly dependent on oil source and fatty acid composition. Unsaturated fatty acids (UFAs) and medium-chain fatty acids (MFAs) may differentially influence microbial populations and fermentation pathways [10]. Plant-derived oils, such as RBO and Palm oil (PO), contain varying proportions of saturated fatty acids (SFAs) and UFAs, which can modify the acetate-to-propionate ratio in the rumen and consequently affect energy utilization efficiency and nutrient digestibility [11]. RBO contains predominantly unsaturated fatty acids, with oleic acid (C18:1) and linoleic acid (C18:2) as major components [12], PO is classified as a common vegetable oil with a higher degree of saturation, characterized notably by a relatively high content of palmitic acid (C16:0) [13]. In contrast, insect-derived oils, particularly black soldier fly larvae oil (BSFLO), are rich in MFAs such as lauric acid (C12:0), which has been reported to alter rumen microbial community structure and potentially improve fermentation efficiency and nutrient utilization [14]. Thus, PO and RBO provide a useful plant-oil comparator with a different fatty acid profile from BSFLO.

Nekrasov et al. [15] investigated the effects of BSFLO supplementation in dairy cows and reported that inclusion of BSFLO at 10 and 100 g/cow/day in early-lactation cows did not exert negative effects on nutrient digestion. Supplementation increased ruminal volatile fatty acid (VFA) concentrations and milk yield. Moreover, lauric acid present in BSFLO exhibited antimicrobial activity by disrupting microbial lipid membranes. In contrast, an in vitro study by Prachumchai and Cherdthong [16] evaluated the effects of BSFLO and roughage-to-concentrate ratio on rumen fermentation. The results demonstrated that supplementation with BSFLO at 4% of dietary dry matter in a diet with a roughage-to-concentrate ratio (R:C) of 40:60 increased total VFA production and in vitro dry matter digestibility. However, increasing the supplementation level to 6% in the same R:C ratio (40:60) reduced in vitro dry matter and organic matter digestibility, ammonia nitrogen (NH_3_-N) concentration, and protozoal population. Beyond its nutritional characteristics, BSFLO has attracted increasing attention as a sustainable alternative lipid source for animal feeding systems. Black soldier fly larvae (BSFL) can be reared on a wide range of organic by-products and agro-industrial residues, converting low-value biomass into high-value nutrients through a biological upcycling process. This characteristic reduces competition between feed ingredients and human food resources while contributing to circular bioeconomy principles and waste management strategies [17,18].

Despite these potential benefits, supplementation with non-protected oils may exert negative effects on fiber-degrading microorganisms. Free fatty acids can coat feed particles or disrupt microbial cell membranes, thereby reducing fiber digestion and overall feed utilization [19,20]. To mitigate these limitations, rumen fat protection technologies, such as the formation of calcium or potassium soaps (Ca-soap and K-soap), have been developed. These protected lipid forms reduce interactions between dietary fats and rumen microorganisms, allowing lipids to bypass ruminal degradation and be digested and absorbed in the abomasum and small intestine. As a result, dairy cows can utilize dietary lipids more efficiently as an energy source without disrupting rumen fermentation balance [21,22].

Therefore, the selection of oil sources and fat protection strategies is a key factor determining the effects of lipid supplementation on rumen fermentation characteristics, nutrient digestibility, and energy utilization efficiency in dairy cows. Systematic comparative studies remain necessary, particularly those evaluating novel lipid sources such as insect-derived oils in combination with different rumen protection technologies, to optimize feed utilization and improve the sustainability of modern dairy production systems.

Although insect-derived and protected plant oils have each been examined individually, no study has directly compared BSFLO with conventional plant oils (PO and RBO) across multiple protection forms (non-protected, calcium soap, and potassium soap) while distinguishing their direct effects on the rumen microbial population from their effects in the presence of a fermentable substrate. To address this gap, the present study used two complementary in vitro systems to evaluate the effects of oil source (BSFLO, PO, and RBO) and protection form (non-protected, calcium soap, and potassium soap) on rumen fermentation, volatile fatty acid profile, NH_3_-N, and dry matter degradability. The first system, conducted without a basal feed substrate (direct assay), was designed to isolate the direct effects of the oils on the ruminal microbial population, whereas the second system, conducted with a basal substrate (diet-associated assay), was designed to assess their effects within an active fermentation environment.

We hypothesized that protecting BSFLO as calcium or potassium soap would preserve ruminal fermentation—maintaining or increasing total volatile fatty acid production and dry matter degradability relative to non-protected oil—and would shift the fermentation pattern toward propionate, thereby lowering the acetate-to-propionate ratio; and further that the magnitude of these responses would depend on the specific oil source × protection form combination and on the presence of a fermentable substrate.

## 2. Materials and Methods

### 2.1. Location, Animal Ethics, and Preparation of Black Soldier Fly Larvae Oil

This research was conducted at Khon Kaen University (KKU), Faculty of Agriculture, Department of Animal Science, located in Khon Kaen, Thailand. The study adhered to animal ethics guidelines under record number IACUC-KKU-48/67, Committee on 17 June 2024. Black soldier fly larvae oil was provided by Fly Lab Tech Co., Ltd., Bangkok, Thailand. The processing involved sanitizing and drying the larvae to 30% of their original mass weight, followed by oil extraction using a press-defatting technique.

### 2.2. Preparation of Protected Fats (Ca-Soap and K-Soap)

Oils were protected using the saponification technique as Deuel [23] method described by Ramana Reddy et al. [24]. Specifically, 25 kg of oil was heated with 38.75 L of 10% (*w*/*v*) sodium hydroxide solution (NaOH; AGC Vinythai Public Company Limited, Samut Prakan, Thailand) until the fatty acids were fully dissolved. Then, 5.875 kg of calcium chloride (CaCl_2_; QRëC™, Auckland, New Zealand) dissolved in 11.75 L of distilled water was added gradually with continuous stirring to precipitate calcium soap. The resulting product was dried overnight at 105 °C [25,26]. For the preparation of potassium soap (K-soap), potassium chloride (KCl; QRëC™, New Zealand) was used in place of calcium chloride, following a modified version of the same procedure. By dividing into smaller scales. Briefly, 300 g of oil was mixed with 465 mL of 10% (*w*/*v*) NaOH solution. The mixture was subsequently combined with either CaCl_2_ or KCl solution (70.5 g in 140 mL of distilled water). Following the addition of the respective salt solution, the mixtures were continuously stirred for 15 min at 50 °C. Subsequently, the preparations were transferred to an oven and dried at 105 °C until dry. The KCl-treated preparation required approximately one additional day to dry compared with the CaCl_2_-treated preparation.

The chemical preparation of the protected oils was based on alkaline saponification followed by salt conversion. Sodium hydroxide (NaOH) was first used to hydrolyze the ester bonds of the triacylglycerols, producing glycerol and sodium salts of the corresponding fatty acids (RCOONa). This reaction is referred to as saponification and converts the fatty acids from their esterified form into ionic carboxylate salts. Subsequent addition of calcium chloride (CaCl_2_) or potassium chloride (KCl) facilitates conversion of the sodium fatty-acid salts into calcium or potassium soaps, respectively. In the calcium treatment, divalent Ca^2+^ ions associate with two fatty-acid anions to form calcium carboxylates [Ca(RCOO)_2_], whereas monovalent K^+^ forms potassium carboxylates (RCOOK). These reactions also produce sodium chloride as a by-product. However, using KCl as a substitute for CaCl_2_ will not result in immediate precipitation; the mixture must be left to stand at room temperature for 30 min, after which the resulting precipitate is filtered out and dried.

### 2.3. Design of Experiment

A completely randomized design (3 × 3 + 1 Factorial Experiments in CRD) with 3 replications was used, the experimental factor included factor A was 3 sources of oils (BSFLO, palm oil (PO) (Oleen; OLEENCO., Ltd., Bangkok, Thailand) and RBO (King; KING RICE OIL GROUP, Bangkok, Thailand)), factor B was 3 form of oils (non-protected, Ca-soap and K-soap) at 4%DM and a control group, which consisted of (1) No oil added (Control) (2) non protected BSFLO (3) non protected PO (4) non protected RBO (5) BSFLO (Ca-soap) (6) PO (Ca-soap) (7) RBO (Ca-soap) (8) BSFLO (K-soap) (9) PO (K-soap) and (10) RBO (K-soap).

This research was divided into two in vitro fermentation systems. The first system was a direct fermentation assay designed as a simplified mechanistic screening approach to evaluate the direct effects of the oil sources and protection forms on ruminal fluid in the absence of an added feed substrate. This assay was not intended to simulate physiological ruminal fermentation because the rumen naturally contains fermentable dietary substrates. Rather, the absence of an external substrate was used to minimize the confounding effects of feed composition and substrate availability and to allow the direct response of the ruminal microbial environment to the added oils to be examined. The results from this assay were subsequently interpreted in conjunction with those from the diet-associated fermentation assay, in which the same oil treatments were evaluated in the presence of the experimental feed substrate. This experiment consisted of 3 replicates × 10 experiments × 2 observation times at 24 and 48 h and was set up for the analysis of VFA, ruminal pH, and NH_3_-N.

A secondary, diet-associated fermentation assay tested the effect of oil on the rumen fluid when feed factors were involved. This experiment was divided into two groups. The first group consisted of 3 replicates × 10 experiments × 2 observation times at 24 and 48 h and was set up for the analysis of VFA, ruminal pH, NH_3_-N, and in vitro dry matter digestibility (IVDMD). The second group consisted of 3 replicate experiments in ankom bags × 10 experiments and was used for the analysis of abomasal simulated digestion.

The formulation ratios for all treatments of the diet-associated fermentation assay were provided in Table 1. The BSFLO levels used in this study are based on previous research by Prachumchai and Cherdthong [16]. Then, the concentrate and roughage were baked at 60 °C for 48 h with a roughage-to-concentrate ratio of 40:60. The sample was ground through a 1 mm sieve, and 0.5 g of the sample was placed in 50 mL vaccine vials supplemented with protected and unprotected BSFLO, RBO, and PO at 4% of dietary dry matter.

### 2.4. Animals, Ruminal Fluid Collection and In Vitro Gas Production

Ruminal fluid collected from two Thai-Holstein crossbred dairy cows, weighing 450 ± 50 kg, was fed a concentrate diet (18% crude protein and 75% total digestible nutrients) and rice straw as a roughage source ad libitum. The concentrate feed consisted of cassava chips, soybean meal, ground corn, palm kernel meal, rice bran, urea, vitamins, and minerals. Feeding was done twice a day at 8:00 a.m. and 4:00 p.m. with 1.5% of body weight as concentrate, and plenty of clean water.

Ruminal fluid was collected in the morning before feeding using an oral esophageal tube connected to an electric vacuum pump. This approach was selected because the donor cows were non-cannulated, and oral stomach-tube collection provides a practical and minimally invasive method for obtaining ruminal fluid for in vitro fermentation studies. Similar procedures using an esophageal or oral stomach tube connected to an electric vacuum pump have been described for ruminal fluid collection in dairy cattle [27]. Ruminal fluid from two cows is mixed together and then poured into a thermos at 39 °C and kept in an anaerobic state, then transported to the laboratory.

The in vitro gas production technique followed the method of Sommart et al. [28]. Buffer and mineral solutions were prepared in advance and continuously flushed with CO_2_ for at least 1 h before the addition of rumen fluid to maintain anaerobic conditions, indicated by a color change in resazurin from blue to light pink. The solutions were maintained at 39 °C on a magnetic stirrer. Ruminal fluid was collected from donor animals using a 244 cm PVC esophageal tube connected to an electric vacuum pump (maximum pressure 68.9 kPa). The first 200 mL of fluid was discarded to avoid contamination with saliva, and 1000 mL of rumen fluid was then collected and stored at 39 °C in pre-warmed thermos flasks. The fluid was filtered through four layers of cheesecloth under continuous CO_2_ flushing and mixed with the pre-prepared buffer and mineral solution.

For incubation, 40 mL of the mixed rumen fluid was added to the test bottle containing the protected (Ca-soap and K-soap) and non-protected (4% concentration) forms of BSFLO, RBO, and PO, and was incubated at 39 degrees Celsius in an incubator.

### 2.5. Analysis of Volatile Fatty Acids (VFA)

Random samples of each experimental factor were collected after 24 and 48 h of incubation in the oven for analysis of VFA using a modification of the method of Yamamoto-Osaki et al. [29] The rumen fluid was centrifuged, then 0.3 mL of 50% sulfuric acid (H_2_SO_4_; RCl Labscan™, Bangkok, Thailand), 0.8 mL of 2-methylvaleric acid (BLD Pharmatech Ltd., Shanghai, China), and 1.6 mL of diethyl ether (QRëC™, New Zealand) were added. After that, it was centrifuged at 4200 rpm for 10 min, and 1 mL of the separated liquid was aspirated into a 1.5 mL vial and analyzed for VFA using a gas chromatography (GC) instrument (Nexis™ GC-2030 Shimadzu Corporation, Kyoto, Japan), equipped with a flame ionization detector (FID). The GC oven temperature was initially set at 100 °C and increased at a rate of 10 °C/min to a final temperature of 250 °C. These included acetic acid (C2), propionic acid (C3), butyric acid (C4), and valeric acid.

### 2.6. Determination of Ammonia-Nitrogen Concentration and pH Value

After 24 and 48 h of incubation in the oven, one flask from each experimental set was randomly selected, and the pH was measured immediately using a pH meter (HANNA instruments HI 98153 microcomputer pH meter, Woonsocket, RI, USA). After that, the microbial activity in the fermentation process was stopped by adding 4 mL of 1N H_2_SO_4_ (at a ratio of 9 parts liquid to 1 part 1N H_2_SO_4_). After that, it was stored at −20 °C to await the analysis of the final fermentation product, including the concentration of NH_3_-N using a KJELTEC Auto 1030 Analyzer (FOSS, Hillerød, Denmark) according to the method of Bremner and Keeney [30].

### 2.7. Analysis of the Digestibility of Dry Matter (In Vitro Dry Matter Digestibility: IVDMD and Abomasal Simulated Digestion Using the DAISYII Incubator)

After the incubation period for the subsamples is complete, the experimental flasks were immediately transferred to a freezer set at −20 °C. This halts microbial activity, preserving the samples for subsequent analysis. When ready for analysis, the subsamples were removed from the freezer and allowed to thaw. Once thawed, the remaining solid residue was analyzed to determine its dry matter content, and this value was used to calculate the dry matter digestibility according to the following equation:

IVDMD (%) = [(initial sample *×* % DM of sample) − (DM residue − Blank)] × 100/(initial sample × %DM)

Artificial abomasum studies are designed to simulate the true digestion of ruminants, as nutrients that are not digested in the rumen pass into the abomasum and are digested within the abomasum, which is more acidic than the rumen. Preparation of artificial abomasum or the HCL-pepsin solution was modified from the method of Gargallo et al. [31]. In total, 2 L of 0.1 N HCL was prepared and placed in the Daisy II Incubator (ANKOM Technologycorperation, Fairport, NY, USA). The pH was adjusted to 1.9–2.0 using 1 M HCL (Hydrochloric acid; QRëC™, New Zealand) solution or 1 M NaOH solution. In total, 1 g/L of P-7000 pepsin (Pepsin from porcine gastric mucosa powder; Sigma-Aldrich Corperation, St. Louis, MO, USA) was placed in the Daisy II jar containing the pH-adjusted HCL solution. For the simulated abomasal digestion procedure, approximately 0.5 g of sample was weighed into each F57 filter bag. Each bag was securely sealed before being placed in a digestion jar. The sample bag was placed in the Daisy II Incubator; the sample was evenly distributed on both sides of the jar. The simulated abomasal digestion solution was added to each jar at a volume of 2000 mL. Each jar contained 24 sample bags. The samples were incubated in the simulated abomasal digestion solution for 2 h. The rotary switch was turned on to rotate the incubator throughout the incubation period and maintain the temperature at 39.5 °C ± 0.5 °C. When the incubation time reached the specified time, it was removed to store the digestate, and the solution was poured into a waste bottle.

The sample bag was rinsed with cold distilled water until the water was clear and colorless. Then, the sample was placed in a hot air oven at 60 °C for 48 h. The completely dried sample was weighed and recorded for digestion calculations.

Digestibility Calculation

% Abomasal simulated digestion As-Received = 100 − ((W3 − (W1 × C1)) × 100/W2)

% Abomasal simulated digestion DM = 100 − ((W3 − (W1 × C1)) × 100/(W2 × DM)

where

W1 = Weight of F57 bag.
C1 = Empty bag (final dry weight in oven/original empty bag weight).
W2 = Sample weight.
DM = % dry matter (multiply by the decimal equivalent).
W3 = Final bag weight after in vitro measurement.

### 2.8. Statistical Analysis

All experimental data were analyzed using analysis of variance (ANOVA) with a 3 × 3 + 1 factorial arrangement under a completely randomized design (CRD). The general linear model (GLM) procedure in SAS (Version 9.00; SAS Institute Inc., Cary, NC, USA) was used to test the main effects of oil source (A) and protection form (B) and their interaction (A × B). When the oil source × protection form interaction was significant (*p* < 0.05), treatment means were interpreted and compared as individual treatment combinations (simple effects) rather than as pooled main effects; when the interaction was not significant, the main effects of oil source and protection form were interpreted. Differences among treatment means were declared significant at *p* < 0.05, and the single control treatment was compared with the oil-supplemented treatments using an orthogonal contrast. Prior to analysis, the normality of residuals and the homogeneity of variances were confirmed using the Shapiro–Wilk and Levene’s tests, respectively, and both assumptions were met. As in the following equation:

Yijk = μ + Ai + Bj + (AB)ij + eijk

Yijk = Observed value of the experimental unit receiving factor A at i, factor B at j, and replicate at k (where ijk = 1, 2, 3).
μ = Overall mean.
Ai = Influence of oil source (BSFLO, PO, and RBO).
Bj = Influence of oil-protected form (non-protected, Ca-soap, and K-soap).
(AB)ij = Joint influence between oil source and oil-protected form.
eijk = Experimental unit error ~ NID (0, σ^2^).

## 3. Results

### 3.1. Ruminal Fermentation and pH Value

The use of oil supplements in ruminant diets affects the rumen fermentation process, as can be seen from the data in Table 2, the effect of oil on total volatile fatty acid (TVFA) content. The control group was found to have the highest total volatile fatty acid content at all time points (72.58 and 57.83 mmol/L at 24 and 48 h, respectively), with a statistically significant difference (*p* < 0.01). In the oil-supplemented group, the average TVFA was 55.98 and 43.04 mmol/L, with a decrease of 22.87% and 25.57% in TVFA content at 24 and 48 h, respectively, compared to the control group. Because the oil source × protection interaction was significant (*p* < 0.01; Table 2), the results are interpreted as individual treatment combinations rather than as separate oil or protection main effects. In the direct fermentation system, non-protected BSFLO and non-protected PO showed the numerically highest TVFA concentrations at 24 h (64.44 and 62.91 mmol/L, respectively), followed by PO K-soap (60.72 mmol/L) and RBO Ca-soap (59.53 mmol/L); however, these four treatments did not differ significantly from one another (shared superscript letter b), and PO K-soap and RBO Ca-soap also did not differ from non-protected RBO (56.33 mmol/L). BSFLO Ca-soap, BSFLO K-soap, PO Ca-soap, and RBO K-soap had the lowest TVFA concentrations at 24 h (48.92–52.22 mmol/L). At 48 h, RBO K-soap had the highest TVFA concentration among the oil-supplemented treatments (53.70 mmol/L), which was significantly higher than that of all other oil treatments but remained lower than that of the control.

Supplementation of oils with basal diet did not exert negative effects on volatile fatty acid production. As shown in Table 2, TVFA concentration at 24 h of incubation was higher in the oil-supplemented treatments compared with the control. Among the individual oil treatments, BSFLO Ca-soap and BSFLO K-soap showed the numerically highest TVFA concentrations (127.02 and 125.87 mmol/L, respectively); however, these values were not significantly different from treatments with overlapping superscript letters, including RBO Ca-soap (117.53 mmol/L) and RBO K-soap (114.84 mmol/L). PO Ca-soap showed the numerically lowest TVFA concentration among the oil treatments (99.10 mmol/L), although it did not differ significantly from non-protected BSFLO (102.89 mmol), non-protected PO (108.78 mmol/L), PO K-soap (110.66 mmol/L), or non-protected RBO (108.97 mmol/L). Consistent with the significant oil × protection interaction, the effect of protection form on TVFA depended on the oil source: calcium soap significantly increased TVFA in BSFLO compared with non-protected BSFLO (127.02 vs. 102.89 mmol/L), whereas it did not significantly change TVFA in PO (99.10 vs. 108.78 mmol). At 48 h of incubation, no significant differences in TVFA concentration were detected between the control and oil-supplemented treatments (*p* = 0.61; control vs. oil-supplemented contrast). Within the oil-supplemented groups, non-protected PO (124.72 mmol) and non-protected RBO (123.84 mmol/L) showed the numerically highest TVFA concentrations, whereas BSFLO K-soap showed the lowest value (99.29 mmol/L), which was significantly lower than that of all other oil treatments except PO Ca-soap (109.96 mmol/L).

Ammonia nitrogen (NH_3_-N) concentrations differed significantly between the control and oil-supplemented treatments. At 24 h of incubation, total NH_3_-N production in the oil-supplemented groups was significantly lower than that in the control group (*p* < 0.05). Among the oil-supplemented treatments, PO Ca-soap showed the numerically lowest NH_3_-N concentration (4.67 mg/dL), whereas RBO K-soap showed the numerically highest value (6.82 mg/dL). PO Ca-soap was significantly lower than all other oil treatments except BSFLO Ca-soap (5.60 mg/dL), whereas RBO K-soap was significantly higher only than PO Ca-soap and BSFLO Ca-soap and did not differ from the remaining oil treatments (5.88–6.17 mg/dL). After 48 h of incubation, NH_3_-N concentration in the control group was significantly lower than that in the oil-supplemented groups (*p* < 0.01). Within the oil treatments, K-soap of RBO again exhibited the highest NH_3_-N concentration (7.75 mg/dL), which was significantly higher than that of all other oil treatments. However, the remaining oil treatments shared overlapping superscript letters, indicating that these numerical differences were not significant in all pairwise comparisons. Because the oil source × protection interaction was significant at both incubation times (*p* < 0.01; Table 2), NH_3_-N responses are best interpreted as treatment combinations rather than as a pooled protection effect; potassium soap generally produced higher NH_3_-N than calcium soap, but the magnitude of this difference depended on the oil source.

Oils supplementation with basal diet showed that NH_3_-N concentrations did not differ between the control and oil-supplemented treatments at either incubation time. In this system, the oil source × protection interaction was not significant at 24 h (*p* = 0.09) but was significant at 48 h (*p* < 0.01; Table 2); accordingly, oil source and protection effects are interpreted as main effects at 24 h and as treatment combinations at 48 h. Within the oil-supplemented groups at 24 h, NH_3_-N concentration did not differ between BSFLO (14.79 mg/dL) and PO (14.51 mg/dL), whereas RBO resulted in a higher NH_3_-N concentration (16.63 mg%; *p* < 0.01). Moreover, oil form significantly influenced NH_3_-N production (*p* < 0.01), with K-soap resulting in a higher NH_3_-N concentration (16.84 mg%) than the non-protected (14.17 mg/dL) and Ca-soap (14.91 mg/dL) forms, which did not differ from each other.

At 48 h of incubation, NH_3_-N concentrations were generally higher in the BSFLO and PO treatments than in the RBO treatments. BSFLO Ca-soap showed the numerically highest NH_3_-N concentration (19.89 mg/dL), but it did not differ significantly from non-protected BSFLO, K-soap of BSFLO, non-protected PO, and K-soap of PO, with values of 18.59, 18.12, 18.49, and 17.93 mg/dL, respectively.

Consistent with the non-significant oil source × protection interaction for pH (*p* ≥ 0.24; Table 2), no significant differences in ruminal pH were observed at either incubation time in either in vitro experiment. In the without-basal in vitro experiment, pH values remained within the normal physiological range (6.5–7.0), indicating that supplementation with different oil sources and protection forms did not alter ruminal pH. In the presence of a basal diet, mean pH values were slightly above this range, at 7.10 and 7.12 at 24 and 48 h of incubation, respectively.

### 3.2. Volatile Fatty Acids Profiles and Branched-Chain Volatile Fatty Acid Profiles

From Table 3, changes in VFA profiles differed significantly among the groups. Because the oil source × protection interaction was significant for acetate, propionate, and the acetate-to-propionate ratio at both incubation times (*p* < 0.05; Table 3), these responses are described as treatment combinations rather than as pooled oil or protection main effects. In the direct (without-basal) incubation, the control group showed a high proportion of C2 at 24 and 48 h (74.30% and 71.32%, respectively), which was significantly higher than the mean of the oil-supplemented groups (*p* < 0.01; control vs. oil-supplemented contrast). At 48 h, it differed significantly only from the BSFLO treatments and non-protected RBO. Among the oil-supplemented groups, non-protected PO and RBO in Ca-soap form had relatively high C2 proportions at 24 h (72.26% and 72.03%), followed by PO K-soap (70.33%), while non-protected PO had the highest proportion among the oil treatments at 48 h (72.54%), which was numerically higher than, but not significantly different from, that of the control. In contrast, RBO in K-soap form had the lowest proportion at 24 h (67.85%) (*p* < 0.01) but was not different from BSFLO in Ca-soap and K-soap form. In the diet-associated fermentation system, dietary oil supplementation significantly affected the proportions of ruminal volatile fatty acids; the proportion of C2 decreased in oil-supplemented treatments compared with the control at both 24 and 48 h of incubation, with reductions of 11.56% and 6.04%, respectively. At 24 h, the lowest C2 proportions (55.30–57.23%) were observed in all BSFLO treatments, non-protected PO and RBO Ca-soap, which did not differ significantly from one another, whereas PO Ca-soap (63.80%), PO K-soap (63.68%) and non-protected RBO (62.45%) did not differ from the control (66.59%). At 48 h, the control (64.89%) differed significantly from non-protected BSFLO (56.77%), BSFLO Ca-soap (58.93%), RBO Ca-soap (58.92%), and RBO K-soap (59.21%), but not from BSFLO K-soap (64.03%), the three PO treatments, or non-protected RBO (60.47%). Non-protected BSFLO had the lowest C2 proportion (56.77%) and was significantly lower only than PO K-soap (68.14%), the control, and BSFLO K-soap (64.03%); PO K-soap showed the numerically highest C2 proportion, but it did not differ significantly from the control or BSFLO K-soap.

The control group exhibited the lowest C3 proportion at 24 h (13.12%), which was significantly lower than that of all oil-supplemented treatments, whereas at 48 h its C3 proportion (14.27%) did not differ significantly from those of the PO treatments and RBO Ca-soap. At 24 h, RBO and BSFLO in K-soap form showed the numerically highest C3 proportions (16.85% and 16.23%, respectively); RBO K-soap did not differ from BSFLO K-soap but was significantly higher than all other treatments, whereas BSFLO K-soap did not differ from non-protected BSFLO or BSFLO Ca-soap (15.69% and 15.68%). In addition, the effect of K-soap on C3 in this direct assay depended on the oil source and was significant only for RBO (16.85% vs. 15.10% for non-protected RBO), consistent with the significant oil × protection interaction (*p* < 0.01). At 48 h, non-protected BSFLO had the highest proportion of C3 (18.96%), which was significantly higher than that of all other treatments. In the diet-associated fermentation system, the mean C3 proportion of the control (19.10% and 19.53% at 24 and 48 h, respectively) was significantly lower than that of the oil-supplemented treatments (*p* < 0.01; control vs. oil-supplemented contrast), although at the individual-treatment level the control did not differ significantly from PO K-soap, PO Ca-soap and non-protected RBO at 24 h or from PO K-soap and non-protected PO at 48 h. The mean C3 proportions of the oil-supplemented treatments were 25.53% and 24.43% at 24 and 48 h, respectively. At 24 h, all forms of BSFLO and Ca-soap of RBO showed the numerically highest C3 proportions, with values of 29.39, 28.54, 28.52, and 26.88%, respectively; however, these values did not differ significantly from those of non-protected PO (26.38%) and RBO K-soap (25.67%). At 48 h, relatively high C3 proportions were observed in non-protected BSFLO (28.28%), RBO Ca-soap (27.43%), and RBO K-soap (26.15%), which did not differ significantly from one another, with non-protected BSFLO showing the numerically highest value. Furthermore, in the with-basal system, the protection-form effect on C3 ran opposite to the direct assay, with Ca-soap combinations tending to give higher C3 than K-soap (e.g., Ca-soap RBO, 27.43%), reflecting the significant oil × protection interaction (*p* < 0.01).

The acetic-to-propionic ratio (A:P ratio) was highest in the control group at 24 h (5.67), being significantly higher than that of all oil-supplemented treatments, whereas at 48 h the control (5.00) did not differ significantly from non-protected PO, PO Ca-soap, and RBO Ca-soap. At 24 h, RBO K-soap had the numerically lowest ratio (4.02), which did not differ significantly from BSFLO K-soap (4.21) and BSFLO Ca-soap (4.38), whereas non-protected PO had the highest ratio among the oil treatments (5.06), followed by RBO Ca-soap (4.94). At 48 h, Non-protected BSFLO had the lowest A:P ratios (3.50), while non-protected PO, PO in Ca-soap form, and RBO in Ca-soap form had higher ratios (5.08, 4.84, and 4.77, respectively), which did not differ significantly from the control. In the diet-associated fermentation system, the A:P ratio of the control (3.49 and 3.32 at 24 and 48 h, respectively) was significantly higher than the mean of the oil-supplemented groups (*p* < 0.01), although it did not differ significantly from PO K-soap at either incubation time (3.15 and 3.46) or from BSFLO K-soap, non-protected PO, and PO Ca-soap at 48 h. Among the oil-supplemented treatments, all forms of BSFLO, non-protected PO, and Ca-soap and K-soap of RBO resulted in lower A:P ratios at 24 h, with values of 1.96, 1.96, 1.99, 2.10, 2.10, and 2.30, respectively. The highest A:P ratio among the oil treatments at 24 h was observed in PO K-soap (3.15), which did not differ significantly from non-protected RBO (2.90) and PO Ca-soap (2.85). Similarly, at 48 h, the lower A:P ratios were observed in non-protected and calcium soap forms of BSFLO and in all forms of RBO, with values of 2.07, 2.39, 2.55, 2.30, and 2.27, respectively, whereas PO K-soap showed the highest A:P ratio (3.46), which was significantly higher than those of the other oil treatments.

For other straight- and branched-chain fatty acids (Table 4), the control group had lower levels of normal-butyric acid (n-C4) than the oil-supplemented groups (*p* < 0.01). The oil source × protection interaction was significant for most of these acids (*p* < 0.05; Table 4), so we describe the differences by treatment combination. At 24 h, BSFLO Ca-soap showed the highest n-C4 proportion (11.61%), which did not differ significantly from PO Ca-soap (11.04%) but was significantly higher than that of all other treatments. At 48 h, BSFLO Ca-soap again showed the highest n-C4 proportion (12.34%), which did not differ significantly from BSFLO K-soap and RBO Ca-soap (11.58% each), whereas non-protected PO showed the lowest value (8.44%), which was significantly lower than that of all other treatments, including the control. At 24 h, BSFLO and PO in Ca-soap form had the highest iso-C4 levels, while the control group showed the lowest level (0.65%; *p* < 0.01). At 48 h, non-protected PO showed the lowest iso-C4 proportion (0.78%), which was significantly lower than that of the control (0.91%) and all other oil treatments. For iso-valeric and normal-valeric acids, the oil-supplemented groups showed significantly higher levels than the control (*p* < 0.01), with the highest values generally occurring in the BSFLO combinations; consistent with the significant oil × protection interaction (*p* < 0.01; Table 4), the magnitude of these increases depended on the protection form. As shown in Table 4, in the diet-associated fermentation system, the normal-butyric acid (n-C4) proportion did not differ between the control and the oil-supplemented treatments at 24 h (*p* = 0.73); however, at 48 h, n-C4 proportions were significantly lower in BSFLO K-soap and PO K-soap (8.53% and 8.61%, respectively) than in the control and all other oil treatments. The proportion of iso-butyric acid (iso-C4) differed significantly between the control and oil-supplemented treatments after 24 h of incubation, with oil supplementation increasing the proportion of iso-C4. The highest iso-C4 proportion was observed in K-soap PO and non-protected RBO (1.38% and 1.56%), whereas the lowest values were observed in non-protected BSFLO and calcium soap PO (0.55% and 0.56%, respectively). No significant treatment effects on iso-C4 proportion were detected after 48 h of incubation.

In the diet-associated (with-basal) fermentation system, the normal-valeric acid proportion differed among treatments after 24 h of incubation, with the lower values in non-protected BSFLO and calcium soap of PO (1.06% and 1.03%, respectively). Iso-valeric acid proportion was affected by treatments at both 24 and 48 h of incubation. At 24 h, the oil-supplemented treatments had a higher mean iso-valeric acid proportion than the control (2.49% vs. 1.85%; *p* < 0.01), and differences were also detected among the oil-supplemented groups. K-soap of PO, non-protected RBO, and K-soap RBO exhibited the highest iso-valeric acid proportions (3.52%, 3.54%, and 2.82%, respectively), whereas non-protected BSFLO and Ca-soap of PO resulted in the lowest values (1.24 and 1.12%). In addition, BSFLO treatments exhibited significantly lower iso-valeric acid proportions compared with plant oil treatments (*p* < 0.01). Conversely, after 48 h of incubation, the control group exhibited a significantly higher iso-valeric acid proportion than the oil-supplemented treatments (*p* < 0.05). Iso-valeric acid proportion was numerically lowest in PO K-soap (1.31%), whereas it was numerically highest in BSFLO Ca-soap and PO Ca-soap (2.63% and 2.70%, respectively), which did not differ significantly from the control (2.54%).

### 3.3. In Vitro Degradability and Abomasal Simulated Digestion

As shown in Table 5, the in vitro dry matter degradability (IVDMD) was significantly lower in the oil-supplemented groups than in the control (*p* < 0.01 at 24 h and *p* < 0.05 at 48 h; control vs. oil-supplemented contrast). The oil source × protection interaction was not significant for IVDMD or for abomasal digestibility (*p* ≥ 0.46; Table 5), so oil source and protection form are interpreted as main effects. The control group showed the highest dry matter digestibility after 24 and 48 h of incubation, with values of 64.03% and 68.44%, respectively. In contrast, the oil-supplemented groups had average digestibility values of 59.20% and 65.00% at 24 and 48 h, respectively. When considering the type of oil supplementation, no significant differences in digestibility among the three oil types were observed at either incubation period. Protection form also did not significantly affect IVDMD at 24 h (*p* = 0.18), although non-protected oils showed a numerically lower value (57.83% DM) than the protected forms (59.88% DM). After 48 h of incubation, no significant differences in dry matter digestibility were detected among the oil-supplemented groups or between protection forms. For abomasal simulated digestion, the control group showed no significant differences in dry matter digestibility compared with the oil-supplemented groups. The average dry matter digestibility of the oil-supplemented groups was 71.78% DM, which is not considered very high in the abomasum.

## 4. Discussion

Oil supplementation redirected ruminal microbial metabolism rather than simply suppressing it, with the direction and magnitude of the shift depending on whether a fermentable substrate was present. Accordingly, the effects of oil supplementation on TVFA production differed between the direct and diet-associated fermentation systems, indicating that the response to protected oils was dependent on the presence of a fermentable feed substrate. In the direct fermentation assay, in which no additional feed substrate was provided, oil supplementation was evaluated primarily for its direct effects on the ruminal microbial environment. Under these conditions, the addition of oils did not consistently demonstrate a stimulatory effect on TVFA production. TVFA content tended to decrease as the fermentation time increased from 24 h to 48 h, which was contrary to the expected trend, as TVFA usually increases continuously with increasing fermentation time. This system was designed as a mechanistic screening assay of the direct interaction between the oils and ruminal fluid; it should not be interpreted as a physiological model of ruminal fermentation because rumen microorganisms normally receive fermentable dietary substrates. The decline in TVFA from 24 to 48 h occurred without a significant treatment effect on pH. Therefore, the decrease cannot be attributed to a demonstrated treatment-related fall in pH in this experiment. Batch incubation may instead have involved depletion of readily available fermentable substrates, changes in microbial activity over time, or microbial interconversion and utilization of fermentation intermediates. These explanations remain possible mechanisms because substrate depletion and microbial populations were not measured. Although low, pH can inhibit acid-sensitive fibrolytic bacteria [32]. In some cases, the VFA produced early in the fermentation process may be further utilized by other microorganisms, such as methanogens that convert acetate and hydrogen to methane, further reducing TVFA by 48 h [33]. In addition, the supplementation of oil in the diet resulted in a decrease in the total volatile fatty acid content, as oil, especially unsaturated fatty acids, has antimicrobial effects, thus reducing the number of fibrolytic bacteria. This is consistent with the study of Sun et al. [34], which demonstrated that dietary PUFA manipulation was accompanied by changes in the relative abundances of several ruminal bacterial taxa, including *Lachnospiraceae UCG-006*, *Selenomonas*, and *Butyrivibrio_2*, together with changes in pH and VFA concentrations. Nevertheless, the present study did not include microbial community or abundance measurements; thus, a similar microbial response cannot be confirmed and remains a hypothesis requiring further investigation.

In the diet-associated fermentation assay, supplementation of dietary oils at 4% of dry matter did not adversely affect TVFA production, particularly during the early stage of incubation. At 24 h, oil-supplemented treatments, especially calcium soap and potassium soap forms of BSFLO, resulted in significantly higher TVFA concentrations compared with the control. This indicates that when fermentable substrates are present, lipid supplementation can enhance ruminal fermentation efficiency rather than suppress it. The increased TVFA production observed in protected BSFLO treatments may be attributed to the reduced toxic effects of free fatty acids on rumen microorganisms. Saponification limits the interaction between lipids and microbial cell membranes, allowing fibrolytic and amylolytic bacteria to maintain metabolic activity. In addition, BSFLO contains medium-chain fatty acids (MCFAs), which, when protected, may selectively inhibit methanogens without strongly suppressing fermentative bacteria. This redirection of metabolic hydrogen away from methanogenesis may enhance fermentation end-product formation, leading to higher TVFA accumulation [14,35]. However, the higher TVFA observed with protected BSFLO should not be interpreted as a universal stimulatory effect of oil protection. The response was evident in the diet-associated fermentation assay, whereas the direct fermentation assay did not provide comparable evidence of enhanced TVFA production. The direct assay was intended as a simplified screening system to evaluate the potential direct effects of oils on ruminal fluid and microbial activity, whereas the diet-associated assay evaluated oil effects within a substrate-containing fermentation environment. Consequently, the presence of dietary substrate appears to be an important factor influencing the response to oil protection. To our knowledge, this is among the first in vitro studies to evaluate insect-derived BSFLO alongside conventional plant oils across two protection forms (calcium and potassium soaps) in both substrate-free and substrate-containing systems. The dual-system design demonstrated that the fermentation response to protected oils is strongly context-dependent, and that BSFLO, irrespective of protection form, shifts fermentation toward propionate more effectively than the plant oils without impairing abomasal simulated digestion.

The use of protected oils in the diet is generally expected to minimize the adverse effects of dietary oils on ruminal fermentation. However, this expectation contrasts with the findings of the direct fermentation assay in the present study, in which supplementation with protected oils resulted in a reduction in volatile fatty acid (VFA) concentrations. This observation may be attributed to the ability of protected oils to maintain a relatively stable ruminal pH, which may alter microbial populations and fermentation dynamics, thereby resulting in lower VFA production [36]. In addition, protected oils do not necessarily increase the amount of oil available within the rumen; rather, they facilitate the passage of dietary lipids out of the rumen, where they can undergo digestion and absorption in the small intestine. Consequently, less lipid remains available for ruminal fermentation, which may explain why protected oils do not necessarily enhance VFA production [37]. Furthermore, oils are generally not considered a primary energy source for anaerobic ruminal microorganisms and, at elevated concentrations, may exert antimicrobial effects that alter key microbial metabolic pathways [38]. At 48 h of incubation, differences in TVFA concentrations between the control and oil-supplemented treatments diminished, suggesting that microbial communities adapt to lipid supplementation over time. The lower TVFA concentration observed at 48 h in some oil treatments, such as BSFLO K-soap in the diet-associated assay, may reflect delayed inhibitory effects of free fatty acids on microbial growth and enzyme activity. The time-dependent decline in TVFA observed in the present study extends the findings of Vargas et al. [20], who noted that lipid supplementation can initially stimulate fermentation but suppress TVFA production during prolonged incubation. Overall, the results indicate that oil supplementation at 4% DM, particularly in protected form, does not impair total ruminal fermentation and may even enhance short-term fermentative activity when sufficient dietary substrates are available.

In the direct fermentation assay, oil supplementation significantly reduced NH_3_-N concentration at 24 h compared with the control, whereas no difference between the control and oil-supplemented treatments was detected in the diet-associated assay. This reduction can be attributed to the inhibitory effects of free fatty acids on ruminal proteolytic and deaminating bacteria. Unsaturated and medium-chain fatty acids, particularly lauric acid (C12:0) present in BSFLO, are known to disrupt microbial cell membranes, thereby suppressing proteolysis and amino acid deamination. Consequently, less ammonia is released into the rumen fluid during early incubation. Similar reductions in NH_3_-N concentration following oil supplementation have been reported by Prachumchai and Cherdthong [16] and Matsuba et al. [19], who observed that lipid-induced antimicrobial effects decreased proteolytic activity in the rumen. At 48 h of incubation, NH_3_-N concentration in oil-supplemented treatments exceeded that of the control. By 48 h, this early suppression of deamination appears to diminish as the fermentative activity of the oil-supplemented and control treatments converged over time within the same fermentation system. As microbial adaptation restored proteolytic and deaminating activity, the nitrogen from the protein substrate that had accumulated during the period of early inhibition was progressively deaminated, allowing NH_3_-N in the oil-supplemented treatments to reach or slightly exceed that of the control [39]. Potassium soap-protected oils tended to produce higher NH_3_-N concentrations than calcium soap-protected oils; however, the significant oil source × protection interaction (Table 2) indicates that the size of this difference depended on the oil source rather than being uniform across oils. Potassium soaps are generally more soluble under ruminal conditions than calcium soaps, increasing their dissociation and interaction with rumen microbes. This may enhance microbial turnover and deamination processes during extended incubation, leading to higher NH_3_-N accumulation. Overall, the NH_3_-N concentrations observed in this study remained within or close to the optimal range for microbial protein synthesis (5–20 mg/dL) [40], indicating that oil supplementation at 4% DM did not compromise ruminal nitrogen metabolism.

Oil supplementation decreased the proportion of C2 compared with the control, consistent with Machmüller et al. [41], who reported that adding unsaturated oil to the cattle diet significantly reduced VFA production, particularly C2 and C4, while increasing C3 production. In the direct assay, K-soap RBO and non-protected BSFLO had significantly higher C3 proportions at 24 and 48 h, respectively, indicating a trend towards increased gluconeogenic pathway digestion along with a decreased A:P ratio. This fermentation shift is commonly associated with lower methane production; however, methane was not measured in the present study, so this interpretation remains an inference. The supplementation of RBO and BSFLO reduced C2 production and promoted C3 production. Notably, the propionate-enhancing effect recorded here for BSFLO was comparable to, or greater than, that of the high-PUFA vegetable oils reported by Vargas et al. [20], who found that both linseed oil and sunflower oil increased C3 production and decreased C4 and ammonia production. A study by Jayanegara et al. [14] also reported that the addition of insect oil, specifically from BSFL, reduced CH4 production without changing the VFA concentration. This is consistent with the fact that insect fatty acids are highly saturated and can directly or through the mechanism of hydrogen competition, reduce the number of methanogenic microorganisms. This disruption of methanogens by medium-chain fatty acids may reduce the uptake of hydrogen in the methanogenesis process, which could leave a higher hydrogen content remaining in the rumen that is available for C3 production. This excess hydrogen is then used to form propionate via the succinate and acrylate pathways [35,42]. In line with the report of Machmüller et al. [43] MCFA supplementation resulted in a decrease in the amount of methanogenic microorganisms and an increase in the proportion of C3. The stronger fermentation shift observed with BSFLO relative to PO and RBO can be explained by differences in their fatty acid profiles. BSFLO is characterized by a high proportion of saturated medium-chain fatty acids, predominantly lauric acid (C12:0, approximately 35%) and myristic acid (C14:0, approximately 11%), whereas PO is dominated by the long-chain saturated palmitic acid (C16:0, approximately 41%) and RBO by the unsaturated oleic (C18:1, approximately 37%) and linoleic (C18:2, approximately 32%) acids. Because medium-chain fatty acids inhibit methanogens and protozoa more selectively than long-chain fatty acids, they may redirect metabolic hydrogen toward propionate formation; this mechanism may help explain the greater reduction in the acetate-to-propionate ratio and the higher propionate proportion recorded for BSFLO than for the two plant oils. The fatty acid composition values reported here were obtained from the supplier’s specifications, as the specific oils used in this study were not analyzed in the laboratory; this should be considered when interpreting the mechanistic comparison.

The increase in C4 in the group treated with BSFLO in the direct assay may instead reflect changes in the relative activity of microbial fermentation pathways and the antimicrobial components naturally found in BSFLO (most notably lauric acid) selectively inhibit certain Gram-positive starch-degraders or specialized hydrogen-producers, but the responsible organisms and substrates were not measured. In a study by Albarki et al. [44], it was found that the use of protected oil in the diet reduced the iso-C4 fraction in the rumen fermentation process, which contradicts this experimental work that increased the iso-butyric fraction. The higher values of iso-C4, iso-valeric, and normal-valeric acids are often associated with amino acid fermentation during rumen fermentation. Especially from branched-chain amino acids (BCAA) and other amino acids with specific structures, oils can affect the rumen microflora and stimulate the breakdown of amino acids, which are the precursors of these acids. However, the effect of Ca-soap and K-soap on these acids was not consistent and depended on the fermentation system and incubation time. For iso-valeric acid, for example, protected oils resulted in lower proportions than non-protected oils at 24 h in the direct assay (2.32% and 2.38% vs. 2.51%) but higher proportions at 48 h (3.03% and 3.15% vs. 2.60%). In the diet-associated assay, K-soap increased iso-valeric acid at 24 h (2.83% vs. 2.51%) but decreased it at 48 h (1.59% vs. 2.53%), whereas Ca-soap decreased it at 24 h (2.14%) and did not differ from non-protected oils at 48 h (2.40%). This pattern is consistent with the significant oil source × protection interaction observed for most branched-chain VFAs (Table 4). Iso-C4 is formed by the breakdown of valine, Iso-valeric acid is formed by the breakdown of leucine, and Normal-valeric acid is formed by the breakdown of threonine and hydroxy acids [45]. Although these branched-chain volatile fatty acids are end products of branched-chain amino acid deamination, a process that would be expected to release ammonia, NH_3_-N concentration in the oil-supplemented treatments was in fact lower than in the control at 24 h in the direct assay. This apparent discrepancy can be reconciled on two grounds. First, branched-chain amino acids constitute only a minor fraction of the total amino acid pool undergoing deamination, so the ammonia contributed by this specific pathway is small relative to the overall reduction in bulk proteolysis and deamination caused by the antimicrobial action of free fatty acids. Second, iso-acids, particularly iso-butyric and iso-valeric acids, serve as essential growth factors and carbon skeletons for microbial amino acid biosynthesis by cellulolytic and other rumen bacteria [45]; their increased availability may have stimulated microbial protein synthesis and thereby promoted rapid re-incorporation of the liberated ammonia into microbial biomass. Consequently, the ammonia released during branched-chain amino acid deamination may have been assimilated almost as quickly as it was produced, so that net NH_3_-N concentration remained low despite the elevated branched-chain volatile fatty acid proportions observed in the direct assay.

The IVDMD results revealed a significant decrease in oil-supplemented groups compared with the control, consistent with the inhibitory effects of lipids on fiber-degrading bacteria [46]. Lipids may also affect fiber digestion through physicochemical interactions with the feed substrate. Dietary oils can associate with or coat the surface of feed particles, potentially reducing microbial attachment and limiting access of fibrolytic microorganisms and their extracellular enzymes to the plant cell wall. Because microbial attachment to feed particles is an important prerequisite for efficient degradation of structural carbohydrates, reduced microbial access may contribute to lower fiber degradation independently of direct microbial inhibition [47]. The depression in IVDMD was numerically more pronounced in non-protected oils at 24 h, although the protection effect was not significant (*p* = 0.18); this tendency may be due to the coating of feed particles by free fatty acids and direct antimicrobial action against cellulolytic microbes such as *Ruminococcus albus* and *Fibrobacter succinogenes* [48]. In addition, A meta-analysis by Dai and Faciola [49] indicated that inhibition of ruminal protozoa can be associated with reductions in fiber digestibility, highlighting the contribution of protozoa to the maintenance of ruminal fiber degradation. In contrast, protected oils (Ca-soap and K-soap) maintained numerically higher degradability values, suggesting that saponification may have partly reduced the adverse interactions between lipids and rumen microbes [50]. After 48 h of incubation, differences among treatments in IVDMD became smaller, implying that the microbial community partially adapted to the lipid environment over time. The maintenance of nutrient degradability by the protected oils in the present study corroborates and extends the observations of Riestanti et al. [26] who observed that Ca-soap of PO preserved microbial fermentation without major declines in nutrient degradability. Thus, protecting oils before supplementation may partly alleviate the negative effects of lipids on rumen digestion. In contrast, this study found no difference in digestibility between the use of protected oil and unprotected oil. This is consistent with the study by Albarki et al. [51], which found that the use of protected oil tended to result in reduced in vitro digestibility. The present study showed that oil supplementation at 4% of dietary DM significantly reduced IVDMD compared with the control. This finding differs from previous reports suggesting that dietary oil supplementation at approximately 3–4% of DM can be used without adversely affecting fiber or dry matter digestibility. However, the effect of supplemental oil on digestibility is not necessarily determined by inclusion level alone and may vary according to the oil source, fatty acid characteristics, protection form, and composition of the basal substrate. For example, previous work cited in this study reported that BSFLO at 4% of dietary DM increased IVDMD when used with a diet containing a 40:60 roughage-to-concentrate ratio, whereas increasing the inclusion level to 6% reduced IVDMD and organic matter digestibility [16].

The abomasal simulated digestion assay approximates post-ruminal digestion under acidic conditions but does not fully reproduce intestinal digestion. In the present study, abomasal simulated digestion values did not differ significantly between the control and oil-supplemented treatments, regardless of oil source or protection form. This indicates that dietary oil supplementation at 4% DM did not impair the overall digestibility of nutrients beyond the rumen. The lack of negative effects on abomasal simulated digestion suggests that lipids, whether protected or non-protected, did not interfere with enzymatic digestion in the simulated abomasum. Protected oils are designed to bypass ruminal degradation and be digested and absorbed in the small intestine, thereby contributing additional energy without reducing nutrient availability. The comparable abomasal simulated digestion values among treatments imply that oil supplementation did not hinder pepsin activity or acid-mediated digestion of residual feed components. Furthermore, the slightly lower IVDMD observed in some oil-supplemented treatments did not translate into reduced abomasal simulated digestion, indicating that undigested nutrients escaping ruminal fermentation were effectively digested under abomasal conditions. This supports the concept that fat protection strategies can mitigate ruminal fermentation depression while preserving abomasal simulated digestion under the conditions tested [26,47].

### Study Limitations

Several limitations should be considered when interpreting these findings. First, the experiments were conducted entirely in vitro using batch incubation; although the two-system design distinguished the direct effects of the oils from their effects in the presence of a fermentable substrate, batch culture cannot fully reproduce ruminal dynamics, and the results require confirmation in vivo, where feed intake, passage rate, and animal performance can be assessed. Second, the fatty acid composition of the specific oils was obtained from supplier specifications rather than measured directly, so the fatty-acid-based mechanistic interpretation remains inferential. Third, microbial community, methanogen, and protozoal populations were not quantified, and the proposed changes in microbial activity were therefore inferred from fermentation end-products rather than measured directly. Fourth, only a single inclusion level (4% of dietary dry matter) and a single roughage-to-concentrate ratio were evaluated, leaving dose–response and diet type interactions to be established. Finally, post-ruminal digestion was estimated using a pepsin–HCl abomasal simulation, which approximates but does not fully represent intestinal digestion. In addition, ruminal fluid was pooled from only two donor cows, and the replicates represented incubation replicates rather than independent animals, which limits inference about between-animal variation. The degree of rumen protection achieved by the calcium and potassium soaps was not chemically characterized, so complete salt conversion and protection efficiency could not be confirmed. Future work combining in vivo evaluation, direct microbial and methane measurements, and multiple inclusion levels is needed to confirm the practical value of protected BSFLO for ruminant nutrition.

## 5. Conclusions

Oil supplementation consistently modified volatile fatty acid profiles toward a more glucogenic fermentation pattern, while branched-chain volatile fatty acid responses suggested altered amino acid fermentation without impairing microbial growth factors. BSFLO Ca-soap and K-soap showed the numerically highest TVFA concentrations at 24 h, although they were not significantly different from RBO Ca-soap and K-soap. At 48 h, the overall control-versus-oil contrast was not significant, but BSFLO K-soap had a lower TVFA concentration than the control in the individual treatment comparison. Importantly, abomasal simulated digestion was not affected by oil supplementation, indicating that lipid inclusion at 4% DM did not compromise simulated post-ruminal digestion under the conditions tested. But these responses did not translate into improved IVDMD. IVDMD was significantly reduced by oil supplementation, and protection did not significantly alleviate this reduction. Overall, under these in vitro conditions, oil supplementation, particularly with BSFLO, shifted fermentation patterns toward propionate production in the presence of adequate dietary substrates, without compromising TVFA production; the value of these oils as energy supplements for ruminants remains to be confirmed in vivo. Because the oil source × protection interaction was significant for most fermentation variables, these benefits are best achieved through specific oil–protection combinations, most notably BSFLO Ca-soap and K-soap for TVFA production, rather than through oil source or protection form alone.

## Figures and Tables

**Table 1:** Feed ingredients and chemical composition of concentrate feed.

Ingredient	Treatments (% of Dietary Dry Matter)									
	T1	T2	T3	T4	T5	T6	T7	T8	T9	T10
Cassava chip	35.00	35.00	35.00	35.00	35.00	35.00	35.00	35.00	35.00	35.00
Soybean meal	22.00	22.00	22.00	22.00	22.00	22.00	22.00	22.00	22.00	22.00
Palm kernel meal	15.50	15.50	15.50	15.50	15.50	15.50	15.50	15.50	15.50	15.50
Rice bran	10.00	6.00	6.00	6.00	6.00	6.00	6.00	6.00	6.00	6.00
Corn meal	15.00	15.00	15.00	15.00	15.00	15.00	15.00	15.00	15.00	15.00
Urea	0.50	0.50	0.50	0.50	0.50	0.50	0.50	0.50	0.50	0.50
Dicalcium-phosphate	1.00	1.00	1.00	1.00	1.00	1.00	1.00	1.00	1.00	1.00
^1^ Premix	0.50	0.50	0.50	0.50	0.50	0.50	0.50	0.50	0.50	0.50
Salt	0.50	0.50	0.50	0.50	0.50	0.50	0.50	0.50	0.50	0.50
BSFLO	-	4.00	-	-	-	-	-	-	-	-
RBO	-	-	4.00	-	-	-	-	-	-	-
PO	-	-	-	4.00	-	-	-	-	-	-
BSFLO Ca-soap	-	-	-	-	4.00	-	-	-	-	-
RBO Ca-soap	-	-	-	-	-	4.00	-	-	-	-
PO Ca-soap	-	-	-	-	-	-	4.00	-	-	-
BSFLO K-soap	-	-	-	-	-	-	-	4.00	-	-
RBO K-soap	-	-	-	-	-	-	-	-	4.00	-
PO K-soap	-	-	-	-	-	-	-	-	-	4.00
Chemical composition (%DM)										
DM	97.98	97.04	97.42	96.65	97.09	97.90	97.56	97.24	96.95	97.30
CP	18.74	19.02	18.62	18.75	18.53	18.66	18.48	18.67	18.43	18.60
EE	2.94	6.52	5.95	6.17	3.01	2.95	3.13	3.04	3.26	3.37
NDF	35.71	32.25	33.21	31.25	34.20	33.16	31.49	30.84	31.27	32.24
ADF	11.99	10.62	9.59	10.38	8.91	9.92	10.23	10.16	9.21	10.37

DM = Dry matter, CP = crude protein, EE = ether extract, NDF = neutral-detergent fiber, ADF = acid-detergent fiber. ^1^ minerals and vitamins (per kg): vitamin A = 10,000,000 IU, vitamin E = 70,000 IU, vitamin D = 1,600,000 IU, Fe = 50 g, Zn = 40 g, Mn = 40 g, Co = 0.1 g, Cu = 10 g, Se = 0.1 g, I: 0.5 g. BSFLO = Black soldier fly larvae oil, RBO = rice bran oil, PO = palm oil, Ca-soap = calcium soap, K-soap = potassium soap.

**Table 2:** Effect of oil supplementation on ruminal fermentation after 24 and 48 h of in vitro incubation.

Oil Type	Protection Methods	Total Volatile Fatty Acid (mmol/L)				Ammonia Nitrogen (mg/dL)				Ruminal pH			
		Without Basal Diet.		With Basal Diet		Without Basal Diet.		With Basal Diet		Without Basal Diet		With Basal Diet	
		24 h	48 h	24 h	48 h	24 h	48 h	24 h	48 h	24 h	48 h	24 h	48 h
Control		72.58 ^a^	57.83 ^a^	99.82 ^d^	115.51 ^abc^	6.44 ^ab^	5.88 ^c^	14.48	16.81 ^bcd^	6.83	6.82	7.18	7.12
BSFLO	Non-protect	64.44 ^b^	40.15 ^def^	102.89 ^cd^	114.62 ^abc^	6.07 ^ab^	5.89 ^c^	13.08	18.59 ^ab^	6.82	6.74	7.15	7.13
	Ca-soap	48.92 ^e^	39.64 ^ef^	127.02 ^a^	113.99 ^abc^	5.60 ^bc^	6.26 ^c^	15.13	19.89 ^a^	6.82	6.8	6.98	7.12
	K-soap	49.22 ^e^	38.95 ^f^	125.87 ^a^	99.29 ^d^	5.88 ^ab^	6.35 ^bc^	16.16	18.12 ^abc^	6.89	6.85	7.12	7.13
PO	Non-protect	62.91 ^b^	48.05 ^c^	108.78 ^bcd^	124.72 ^a^	5.97 ^ab^	7.01 ^b^	13.17	18.49 ^ab^	6.95	6.86	7.01	7.13
	Ca-soap	49.54 ^e^	39.92 ^def^	99.10 ^d^	109.96 ^cd^	4.67 ^c^	6.54 ^bc^	14.39	16.35 ^cde^	6.79	6.84	7.12	7.11
	K-soap	60.72 ^bc^	43.16 ^d^	110.66 ^bcd^	112.10 ^c^	6.16 ^ab^	6.35 ^bc^	15.97	17.93 ^abc^	6.76	6.79	7.15	7.15
RBO	Non-protect	56.33 ^cd^	42.87 ^de^	108.97 ^bcd^	123.84 ^ab^	6.16 ^ab^	6.07 ^c^	16.25	15.79 ^de^	6.86	6.85	7.08	7.09
	Ca-soap	59.53 ^bc^	40.92 ^def^	117.53 ^ab^	112.89 ^bc^	6.17 ^ab^	6.08 ^c^	15.23	14.67 ^e^	6.80	6.93	7.10	7.09
	K-soap	52.22 ^de^	53.70 ^b^	114.84 ^abc^	117.20 ^abc^	6.82 ^a^	7.75 ^a^	18.4	16.07 ^cde^	6.89	6.84	7.12	7.16
SEM		1.06	0.70	2.62	2.23	0.21	0.14	0.53	0.43	0.06	0.05	0.06	0.06
Oil × Protection (interaction)		*p* < 0.01	*p* < 0.01	*p* < 0.01	*p* < 0.01	*p* < 0.01	*p* < 0.01	0.09	*p* < 0.01	0.27	0.35	0.25	0.24
Control vs. oil-supplemented		*p* < 0.01	*p* < 0.01	*p* < 0.01	0.61	*p* < 0.05	*p* < 0.01	0.16	0.27	0.81	0.82	0.18	0.61
Control		72.58 ^a^	57.83 ^a^	99.82 ^b^	115.51	6.44 ^a^	5.88 ^b^	14.48	16.81	6.83	6.82	7.18	7.12
Others		55.98 ^b^	43.04 ^b^	112.85 ^a^	114.29	5.95 ^b^	6.48 ^a^	15.31	17.32	6.84	6.83	7.09	7.12
Oil type		*p* < 0.01	*p* < 0.01	*p* < 0.01	*p* < 0.01	*p* < 0.01	*p* < 0.01	*p* < 0.01	*p* < 0.01	0.97	0.17	0.93	0.87
BSFLO		54.19 ^c^	39.58 ^c^	118.59 ^a^	109.30 ^b^	5.85 ^b^	6.16 ^b^	14.79 ^b^	18.87 ^a^	6.84	6.8	7.08	7.13
PO		57.73 ^a^	43.71 ^b^	106.18 ^c^	115.59 ^a^	5.60 ^bc^	6.63 ^a^	14.51 ^b^	17.59 ^b^	6.84	6.83	7.09	7.13
RBO		56.03 ^b^	45.83 ^a^	113.78 ^b^	117.98 ^a^	6.38 ^a^	6.63 ^a^	16.63 ^a^	15.50 ^c^	6.85	6.88	7.1	7.11
Protection methods		*p* < 0.01	*p* < 0.01	*p* < 0.01	*p* < 0.01	*p* < 0.01	*p* < 0.01	*p* < 0.01	0.21	0.33	0.66	0.37	0.39
Non-protected		61.23 ^a^	43.69 ^b^	106.88 ^c^	121.06 ^a^	6.07 ^ab^	6.32 ^b^	14.17 ^b^	17.62	6.88	6.82	7.08	7.12
Ca-soap		52.66 ^c^	40.16 ^c^	114.55 ^b^	112.28 ^b^	5.48 ^b^	6.29 ^b^	14.91 ^b^	16.97	6.8	6.86	7.06	7.11
K-soap		54.05 ^b^	45.27 ^a^	117.12 ^a^	109.53 ^c^	6.29 ^a^	6.82 ^a^	16.84 ^a^	17.37	6.85	6.83	7.13	7.15

^a–f^ = different superscript letters in the same column indicate significant differences among treatments (*p* < 0.05). BSFLO = Black soldier fly larvae oil; PO = palm oil; RBO = rice bran oil; Ca-soap = calcium soap; K-soap = potassium soap; SEM = standard error of the mean.

**Table 3:** Effect of oil supplementation on volatile fatty acid profiles after 24 and 48 h of in vitro incubation.

Oil Type	Protection Methods	Acetic Acid (%TVFA)				Propionic Acid (%TVFA)				Acetic: Propionic Ratio			
		Without Basal Diet		With Basal Diet		Without Basal Diet		With Basal Diet		Without Basal Diet		With Basal Diet	
		24 h	48 h	24 h	48 h	24 h	48 h	24 h	48 h	24 h	48 h	24 h	48 h
Control		74.30 ^a^	71.32 ^ab^	66.59 ^a^	64.89 ^ab^	13.12 ^e^	14.27 ^e^	19.10 ^d^	19.53 ^e^	5.67 ^a^	5.00 ^a^	3.49 ^a^	3.32 ^ab^
BSFLO	Non-protect	69.23 ^c^	66.39 ^c^	57.23 ^cd^	56.77 ^d^	15.69 ^bc^	18.96 ^a^	29.39 ^a^	28.28 ^a^	4.41 ^de^	3.50 e	1.96 ^d^	2.07 ^c^
	Ca-soap	68.67 ^cd^	65.94 ^c^	55.84 ^d^	58.93 ^cd^	15.68 ^bc^	15.76 ^bcd^	28.54 ^a^	24.66 ^bc^	4.38 ^def^	4.08 ^d^	1.96 ^d^	2.39 ^c^
	K-soap	68.24 ^cd^	66.88 ^c^	56.62 ^d^	64.03 ^abc^	16.23 ^ab^	15.88 ^bc^	28.52 ^a^	23.45 ^c^	4.21 ^ef^	4.13 ^d^	1.99 ^d^	2.73 ^bc^
PO	Non-protect	72.26 ^b^	72.54 ^a^	55.30 ^d^	61.67 ^bcd^	14.30 ^d^	14.29 ^e^	26.38 ^ab^	23.07 ^cde^	5.06 ^b^	5.08 ^a^	2.10 ^d^	2.68 ^bc^
	Ca-soap	69.60 ^c^	68.66 ^bc^	63.80 ^ab^	60.63 ^bcd^	14.97 ^cd^	14.33 ^e^	22.51 ^bcd^	23.25 ^cd^	4.65 ^cd^	4.84 ^a^	2.85 ^bc^	2.66 ^bc^
	K-soap	70.33 ^bc^	68.68 ^bc^	63.68 ^ab^	68.14 ^a^	15.10 ^cd^	15.28 ^cde^	20.22 ^d^	19.80 ^de^	4.66 ^cd^	4.50 ^bc^	3.15 ^ab^	3.46 ^a^
RBO	Non-protect	69.71 ^c^	67.04 ^c^	62.45 ^abc^	60.47 ^bcd^	15.10 ^cd^	15.95 ^bc^	21.60 ^cd^	23.78 ^c^	4.62 ^cd^	4.26 ^cd^	2.90 ^b^	2.55 ^c^
	Ca-soap	72.03 ^b^	68.09 ^bc^	56.12 ^d^	58.92 ^cd^	14.59 ^d^	14.72 ^de^	26.88 ^a^	27.43 ^ab^	4.94 ^bc^	4.77 ^ab^	2.10 ^d^	2.30 ^c^
	K-soap	67.85 ^d^	68.01 ^bc^	58.80 ^bcd^	59.21 ^cd^	16.85 ^a^	16.46 ^b^	25.67 ^abc^	26.15 ^abc^	4.02 ^f^	4.14 ^d^	2.30 ^cd^	2.27 ^c^
SEM		0.33	0.69	1.06	1.07	0.19	0.22	0.87	0.72	0.07	0.06	0.11	0.14
Oil × Protection (interaction)		*p* < 0.01	*p* < 0.05	*p* < 0.01	*p* < 0.01	*p* < 0.01	*p* < 0.01	*p* < 0.01	*p* < 0.01	*p* < 0.01	*p* < 0.01	*p* < 0.01	*p* < 0.01
Control vs. oil-supplemented		*p* < 0.01	*p* < 0.01	*p* < 0.01	*p* < 0.01	*p* < 0.01	*p* < 0.01	*p* < 0.01	*p* < 0.01	*p* < 0.01	*p* < 0.01	*p* < 0.01	*p* < 0.01
Control		74.30 ^a^	71.32 ^ab^	66.59 ^a^	64.89 ^ab^	13.12 ^b^	14.27 ^b^	19.10 ^b^	19.53 ^b^	5.67 ^a^	5.00 ^a^	3.49 ^a^	3.32 ^a^
Others		69.76 ^b^	68.02 ^b^	58.87 ^b^	60.97 ^b^	15.39 ^a^	15.73 ^a^	25.53 ^a^	24.43 ^a^	4.35 ^b^	4.37 ^b^	2.37 ^b^	2.57 ^b^
Oil type		*p* < 0.01	*p* < 0.01	*p* < 0.01	*p* < 0.01	*p* < 0.01	*p* < 0.01	*p* < 0.01	*p* < 0.01	*p* < 0.01	*p* < 0.01	*p* < 0.01	*p* < 0.01
BSFLO		68.71 ^b^	66.40 ^c^	56.56 ^b^	59.91 ^b^	15.87 ^a^	16.86 ^a^	28.82 ^a^	25.47 ^a^	4.33 ^b^	3.90 ^c^	1.97 ^b^	2.40 ^b^
PO		70.73 ^a^	69.96 ^a^	60.93 ^a^	63.48 ^a^	14.79 ^b^	14.64 ^c^	23.04 ^b^	22.04 ^b^	4.79 ^a^	4.81 ^a^	2.70 ^a^	2.93 ^a^
RBO		69.86 ^a^	67.72 ^b^	59.13 ^a^	59.53 ^b^	15.51 ^a^	15.71 ^b^	24.72 ^b^	25.79 ^a^	4.53 ^ab^	4.39 ^b^	2.43 ^a^	2.37 ^b^
Protection methods		*p* < 0.01	0.19	0.29	*p* < 0.01	*p* < 0.01	*p* < 0.01	0.25	*p* < 0.01	*p* < 0.01	*p* < 0.01	0.15	*p* < 0.01
Non-protected		70.40 ^a^	68.66	58.33	59.64 ^b^	15.03 ^b^	16.40 ^a^	25.79	25.05 ^a^	4.70 ^a^	4.28 ^b^	2.32	2.43 ^b^
Ca-soap		70.10 ^a^	67.56	58.59	59.49 ^b^	15.08 ^b^	14.94 ^c^	25.98	25.11 ^a^	4.66 ^a^	4.57 ^a^	2.30	2.45 ^b^
K-soap		68.80 ^b^	67.86	59.70	63.79 ^a^	16.06 ^a^	15.87 ^b^	24.81	23.13 ^b^	4.30 ^b^	4.25 ^b^	2.48	2.82 ^a^

^a–f^ = different superscript letters in the same column indicate significant differences among treatments (*p* < 0.05). BSFLO = Black soldier fly larvae oil; PO = palm oil; RBO = rice bran oil; Ca-soap = calcium soap; K-soap = potassium soap; SEM = standard error of the mean.

**Table 4:** Effect of oil supplementation on other straight-chain volatile fatty acid and branched-chain volatile fatty acid profiles after 24 and 48 h of in vitro incubation.

Oil Type	Protection Methods	Normal-Butyric Acid (%TVFA)				Iso-Butyric Acid (%TVFA)				Normal-Valeric Acid (%TVFA)				Iso-Valeric Acid (%TVFA)			
		Without Basal Diet		With Basal Diet		Without Basal Diet		With Basal Diet		Without Basal Diet		With Basal Diet		Without Basal Diet		With Basal Diet	
		24 h	48 h	24 h	48 h	24 h	48 h	24 h	48 h	24 h	48 h	24 h	48 h	24 h	48 h	24 h	48 h
Control		8.97 ^d^	10.00 ^d^	10.31 ^ab^	11.85 ^a^	0.65 ^f^	0.91 ^d^	0.66 ^de^	0.87	0.86 ^f^	1.02	1.37 ^bc^	1.34	2.17 ^de^	2.47 ^e^	1.85 ^cd^	2.54 ^abc^
BSFLO	Non-protect	10.55 ^b^	11.02 ^bc^	10.27 ^ab^	11.16 ^a^	0.78 ^cd^	0.94 ^d^	0.55 ^e^	0.76	1.07 ^cd^	1.40	1.06 ^c^	1.26	2.74 ^a^	2.73 ^d^	1.24 ^de^	2.43 ^abcd^
	Ca-soap	11.61 ^a^	12.34 ^a^	10.36 ^ab^	11.72 ^a^	0.89 ^a^	1.18 ^a^	1.11 b^c^	0.70	1.16 ^b^	1.44	1.54 ^ab^	1.18	2.50 ^b^	3.15 ^a^	2.73 ^b^	2.63 ^a^
	K-soap	10.85 ^b^	11.58 ^ab^	10.19 ^ab^	8.53 ^b^	0.83 ^bc^	1.10 ^abc^	0.99 ^c^	0.76	1.35 ^a^	1.54	1.52 ^ab^	1.46	2.56 ^ab^	3.17 ^a^	2.16 ^bc^	1.77 ^cde^
PO	Non-protect	9.54 ^cd^	8.44 ^e^	12.91 ^a^	10.82 ^a^	0.75 ^de^	0.78 ^e^	1.08 ^bc^	0.76	0.94 ^ef^	0.98	1.56 ^ab^	1.15	2.39 ^bc^	2.20 ^f^	2.76 ^b^	2.54 ^abc^
	Ca-soap	11.04 ^ab^	11.27 ^b^	10.64 ^ab^	11.58 ^a^	0.88 ^ab^	1.08 ^bc^	0.56 ^e^	0.85	1.12 ^bc^	1.27	1.03 ^c^	1.20	2.39 ^bc^	2.94 ^bc^	1.12 ^e^	2.70 ^a^
	K-soap	10.40 ^b^	10.23 ^cd^	9.39 ^b^	8.61 ^b^	0.81 ^c^	1.16 ^ab^	1.38 ^ab^	0.80	1.02 ^de^	1.28	1.80 ^ab^	1.31	2.36 ^bcd^	3.10 ^ab^	3.52 ^a^	1.31 ^e^
RBO	Non-protect	10.88 ^b^	11.07 ^bc^	9.19 ^b^	11.03 ^a^	0.81 ^c^	1.07 ^c^	1.56 ^a^	0.82	1.05 ^cd^	1.23	1.92 ^a^	1.29	2.41 ^b^	2.86 ^cd^	3.54 ^a^	2.61 ^ab^
	Ca-soap	9.64 ^c^	11.58 ^ab^	11.70 ^ab^	11.18 ^a^	0.71 ^e^	1.16 ^ab^	1.19 ^bc^	0.77	0.96 ^e^	1.31	1.66 ^ab^	1.17	2.06 ^e^	3.00 ^abc^	2.56 ^bc^	1.86 ^bcde^
	K-soap	10.91 ^b^	10.00 ^d^	10.44 ^ab^	10.90 ^a^	0.82 ^c^	1.19 ^a^	0.88 ^cd^	0.80	1.28 ^a^	1.29	1.38 ^bc^	1.26	2.21 ^cde^	3.17 ^a^	2.82 ^ab^	1.69 ^de^
SEM		0.13	0.18	0.68	0.35	0.01	0.02	0.06	0.07	0.02	0.06	0.09	0.09	0.04	0.04	0.15	0.15
Oil × Protection (interaction)		*p* < 0.01	*p* < 0.01	*p* < 0.05	*p* < 0.01	*p* < 0.01	*p* < 0.01	*p* < 0.01	0.77	*p* < 0.01	0.25	*p* < 0.01	0.65	*p* < 0.01	*p* < 0.01	*p* < 0.01	*p* < 0.01
Control vs. oil-supplemented		*p* < 0.01	*p* < 0.01	0.73	*p* < 0.01	*p* < 0.01	*p* < 0.01	*p* < 0.01	0.23	*p* < 0.01	*p* < 0.01	0.20	0.38	*p* < 0.01	*p* < 0.01	*p* < 0.01	*p* < 0.05
Control		8.97 ^b^	10.00 ^b^	10.31	11.85 ^a^	0.65 ^b^	0.91 ^b^	0.66 ^b^	0.87	0.86 ^b^	1.02 ^b^	1.37	1.34	2.17 ^b^	2.47 ^b^	1.85 ^b^	2.54 ^a^
Others		10.60 ^a^	10.84 ^a^	10.56	10.61 ^b^	0.81 ^a^	1.07 ^a^	1.03 ^a^	0.78	1.11 ^a^	1.30 ^a^	1.50	1.25	2.40 ^a^	2.92 ^a^	2.49 ^a^	2.17 ^b^
Oil type		*p* < 0.01	*p* < 0.01	0.43	0.06	*p* < 0.01	*p* < 0.01	*p* < 0.01	0.45	*p* < 0.01	*p* < 0.05	*p* < 0.01	0.57	*p* < 0.01	*p* < 0.01	*p* < 0.01	0.21
BSFLO		11.00 ^a^	11.65 ^a^	10.27	10.47	0.83 ^a^	1.07 ^b^	0.88 ^c^	0.74	1.19 ^a^	1.46 ^a^	1.38 ^c^	1.30	2.60 ^a^	3.02 ^a^	2.04 ^c^	2.28
PO		10.33 ^b^	9.98 ^c^	10.98	10.34	0.81 ^a^	1.01 ^b^	1.01 ^b^	0.80	1.03 ^b^	1.18 ^c^	1.46 ^b^	1.22	2.38 ^b^	2.77 ^b^	2.47 ^b^	2.18
RBO		10.48 ^b^	10.88 ^b^	10.44	11.04	0.78 ^b^	1.14 ^a^	1.21 ^a^	0.80	1.10 ^a^	1.28 ^b^	1.65 ^a^	1.24	2.23 ^b^	3.01 ^a^	2.97 ^a^	2.05
Protection methods		*p* < 0.01	*p* < 0.01	0.24	*p* < 0.01	*p* < 0.01	*p* < 0.01	*p* < 0.05	0.97	*p* < 0.01	*p* < 0.01	*p* < 0.01	0.13	*p* < 0.01	*p* < 0.01	*p* < 0.01	*p* < 0.01
Non-protected		10.32 ^b^	10.18 ^b^	10.79	11.00 ^a^	0.78 ^b^	0.93 ^b^	1.06 ^a^	0.78	1.02 ^b^	1.21 ^b^	1.51 ^a^	1.23	2.51 ^a^	2.60 ^b^	2.51 ^b^	2.53 ^a^
Ca-soap		10.76 ^a^	11.73 ^a^	10.90	11.49 ^a^	0.83 ^a^	1.14 ^a^	0.95 ^b^	0.76	1.08 ^b^	1.34 ^a^	1.41 ^b^	1.18	2.32 ^b^	3.03 ^a^	2.14 ^c^	2.40 ^aa^
K-soap		10.72 ^a^	10.60 ^b^	10.01	9.34 ^b^	0.82 ^a^	1.15 ^a^	1.08 ^a^	0.79	1.22 ^a^	1.37 ^a^	1.57 ^a^	1.34	2.38 ^b^	3.15 ^a^	2.83 ^a^	1.59 ^b^

^a–f^ = different superscript letters in the same column indicate significant differences among treatments (*p* < 0.05). BSFLO = Black soldier fly larvae oil; PO = palm oil; RBO = rice bran oil; Ca-soap = calcium soap; K-soap = potassium soap; SEM = standard error of the mean.

**Table 5:** Effect of oil supplementation on in vitro dry matter degradability (IVDMD) in rumen and abomasal-simulated digestion.

Oil Type	Protection Methods	IVDMD (%DM)		Abomasal Simulated Digestion (%DM)
		24 h	48 h	
Control		64.03	68.44	72.22
BSFLO	Non-protect	56.02	65.08	72.10
	Ca-soap	61.15	66.08	71.48
	K-soap	57.51	64.77	72.29
PO	Non-protect	59.89	63.50	71.85
	Ca-soap	60.37	63.91	71.62
	K-soap	59.99	65.91	71.73
RBO	Non-protect	57.57	63.79	71.21
	Ca-soap	59.47	66.71	71.39
	K-soap	60.83	65.20	72.39
SEM		1.53	1.20	0.61
Oil × Protection (interaction)		0.46	0.47	0.84
Control vs. oil-supplemented		*p* < 0.01	*p* < 0.05	0.51
Control		64.03 ^a^	68.44 ^a^	72.22
Others		59.20 ^b^	65.00 ^b^	71.78
Oil type		0.38	0.60	0.84
BSFLO		58.23	65.31	71.96
PO		60.08	64.44	71.73
RBO		59.29	65.23	71.66
Protection methods		0.18	0.29	0.46
Non-protected		57.83	64.13	71.72
Ca-soap		60.33	65.57	71.51
K-soap		59.44	65.29	72.14

^a,b^ = different superscript letters in the same column indicate significant differences among treatments (*p* < 0.05). BSFL oil = Black soldier fly larvae oil; IVDMD = in vitro dry matter degradability; DM = dry matter; SEM = standard error of the mean.

## Data Availability

The raw data supporting the conclusions of this article will be made available by the authors on request.

## Abbreviations

The following abbreviations are used in this manuscript:

BSFLO	Black soldier fly larvae oil
C2	Acetate
C3	Propionate
C4	Butyrate
Ca-soap	Calcium soap
GC	Gas chromatograph
IVDMD	In vitro dry matter degradability
ASD	Abomasal simulated digestion
K-soap	Potassium
NH_3_-N	Ammonia nitrogen
PO	Palm oil
RBO	Rice bran oil
SEM	Standard error of the mean
VFA	Volatile fatty acid
